# The Relationship between Colonization by *Moraxella catarrhalis* and Tonsillar Hypertrophy

**DOI:** 10.1155/2018/5406467

**Published:** 2018-11-01

**Authors:** Mirela C. M. Prates, Edwin Tamashiro, José L. Proenca-Modena, Miriã F. Criado, Tamara H. Saturno, Anibal S. Oliveira, Guilherme P. Buzatto, Bruna L. S. Jesus, Marcos G. Jacob, Lucas R. Carenzi, Ricardo C. Demarco, Eduardo T. Massuda, Davi Aragon, Fabiana C. P. Valera, Eurico Arruda, Wilma T. Anselmo-Lima

**Affiliations:** ^1^Department of Ophthalmology, Otorhinolaryngology, Head and Neck Surgery, Ribeirao Preto Medical School, University of São Paulo, Ribeirao Preto, SP 14049-900, Brazil; ^2^Department of Cell Biology, Ribeirao Preto Medical School, University of São Paulo, Ribeirao Preto, SP 14049-900, Brazil; ^3^Virology Research Center, Ribeirao Preto Medical School, University of São Paulo, Ribeirao Preto, SP 14049-900, Brazil; ^4^Department of Genetics, Evolution and Bioagents, Institute of Biology, University of Campinas, Campinas, SP 13083-970, Brazil; ^5^Department of Pediatrics, Ribeirao Preto Medical School, University of São Paulo, Ribeirao Preto, SP 14049-900, Brazil

## Abstract

We sought to investigate the prevalence of potentially pathogenic bacteria in secretions and tonsillar tissues of children with chronic adenotonsillitis hypertrophy compared to controls. Prospective case-control study comparing patients between 2 and 12 years old who underwent adenotonsillectomy due to chronic adenotonsillar hypertrophy to children without disease. We compared detection of *Streptococcus pneumoniae*, *Haemophilus influenzae*, *Staphylococcus aureus, Pseudomonas aeruginosa*, and *Moraxella catarrhalis* by real-time PCR in palatine tonsils, adenoids, and nasopharyngeal washes obtained from 37 children with and 14 without adenotonsillar hypertrophy. We found high frequency (>50%) of *Haemophilus influenzae*, *Streptococcus pneumoniae*, *Moraxella catarrhalis*, and *Pseudomonas aeruginosa* in both groups of patients. Although different sampling sites can be infected with more than one bacterium and some bacteria can be detected in different tissues in the same patient, adenoids, palatine tonsils, and nasopharyngeal washes were not uniformly infected by the same bacteria. Adenoids and palatine tonsils of patients with severe adenotonsillar hypertrophy had higher rates of bacterial coinfection. There was good correlation of detection of *Moraxella catarrhalis* in different sampling sites in patients with more severe tonsillar hypertrophy, suggesting that *Moraxella catarrhalis* may be associated with the development of more severe hypertrophy, that inflammatory conditions favor colonization by this agent. *Streptococcus pneumoniae*, *Staphylococcus aureus*, *Haemophilus influenzae*, and *Moraxella catarrhalis* are frequently detected in palatine tonsils, adenoids, and nasopharyngeal washes in children. Simultaneous detection of *Moraxella catarrhalis* in adenoids, palatine tonsils, and nasopharyngeal washes was correlated with more severe tonsillar hypertrophy.

## 1. Introduction

Chronic tonsillar diseases have great impact in general health worldwide, particularly in children [1]. Chronic inflammation and hypertrophy of the tonsils may be associated with complications such as chronic rhinosinusitis, auditory tube dysfunction, otitis media, obstructive sleep apnea, periodontal problems, and alterations in orofacial and behavioral development in children [2–4]. Despite the great public health importance of chronic adenotonsillar hypertrophy, little is known of its pathogenesis.

Multiple factors are involved in host colonization by microorganisms [5]. The human microbiota creates a microenvironment that allows for interactions between different microorganisms [6], and evidence suggests that infections by different combinations of microorganisms may lead to increased adenotonsillar sizes [7–9].

Bacteria such as *Streptococcus pneumoniae (S. pneumoniae)*, *Haemophilus influenzae (H. influenzae)*, *Staphylococcus aureus* (*S. aureus*), and *Moraxella catarrhalis (M. catarrhalis)* are commonly detected in the upper respiratory tract of healthy individuals and also in acute upper respiratory diseases [10–13], but it is still unclear if they have any pathogenic role in chronic adenotonsillitis. These bacteria can potentially interact with each other in biofilms and stimulate chronic tonsillar inflammation [14, 15]. One study evaluated the possible effect of biofilm-producing bacteria (BPB) in tonsillar specimens in clinical features of 22 children, and they observed a significant correlation of BPB presence with intensity of tonsillar hyperplasia, being *S. aureus* the most frequent pathogen.

In the present study, the presence of genomic DNA of five bacteria commonly detected in respiratory infections was associated with symptoms and signs of chronic adenotonsillar disease.

## 2. Material and Methods

### 2.1. Study Design

In this cross-sectional case-control study, the frequency of detection of bacterial genomes was compared between children with chronic adenotonsillar hypertrophy and controls. The study was conducted between May 2010 and August 2012, with children 2–12 years old (mean of 6 years) who were treated at the Otorhinolaryngology Division of the University Hospital, Medical School of Ribeirao Preto, University of São Paulo.

The study group consisted of children who underwent adenotonsillectomy due to chronic adenotonsillar hypertrophy. Before surgery, all children were assessed with a full otorhinolaryngological examination, including flexible nasal endoscopy. Inclusion criteria were the presence of sleep apnea, tonsillar hypertrophy grade ≥3 (both palatine tonsils (PT) occupied 50% or more of the oropharynx width), and the adenoid (AD) occupied 70% or more of the nasopharynx at endoscopy.

The control group consisted of children who underwent cochlear implantation, in the absence of symptoms of adenotonsillar disease or hypertrophy, palatine tonsils graded ≤2 (both PT occupied 50% or less of the oropharynx width), and AD occupied 50% or less of the nasopharynx at endoscopy.

Exclusion criteria for both groups were the presence of symptoms and signs of acute infection of the upper respiratory airways at the time of surgery, antibiotic use 4 weeks prior to surgery, past history of adenotonsillar or sinonasal surgery, genetic syndromes, a history suggestive of primary ciliary dyskinesia, cystic fibrosis, or immunodeficiencies.

During adenotonsillectomy under general anesthesia, three samples were collected from every patient: (a) nasopharyngeal wash (NPW): saline flushed into both nasal cavities and collected at the nasopharynx using a sterile syringe; (b) fragment of adenoid tissue (AD), collected with a conventional Beckman curette for adenoidectomy; and (c) fragment of palatine tonsil (PT) collected with a cold-knife scalpel. From control patients, during surgery for cochlear implant, a mouth-opener was placed and small tissue fragments were obtained with punch biopsy from adenoid and palatine tonsil tissues, apart from NPW.

Tissue samples from PT and AD were placed in Eagle's minimal essential medium (MEM) supplemented with 10% fetal bovine serum and 15% antibiotic-antimycotic solution (penicillin-streptomycin 20,000 U/ml and amphotericin B 200 mg/mL, both from Gibco (Grand Island, NY, USA)) and transported on ice within a maximum of 4 hours for further processing in the laboratory. Tissue samples were washed twice with MEM to remove debris and blood clots, and then minced in TRIzol® reagent (Invitrogen, Carlsbad, CA, USA) for subsequent extraction of total nucleic acids. NPW samples were distributed in several aliquots, including one of 250 *µ*L added to 750 *µ*L of TRIzol®. All samples were frozen at −70°C until further testing.

### 2.2. Detection of Bacterial DNA

The bacterial genome was detected by real-time PCR using TaqMan probes (Applied Biosystems®, Foster City, CA, USA) after DNA extraction with TRIzol®. Primers and probes were designed to detect *S. aureus*, *S. pneumoniae*, *H. influenzae*, *M. catarrhalis*, and *Pseudomonas aeruginosa* (*P. aeruginosa*) (Table 1). Each real-time PCR assay was done with 1 *µ*L of extracted DNA (approximately 100 ng), 0.25 *µ*L of specific *primers* (10 pmol/*µ*L), 0.125 *µ*L of probe (10 pmol/*µ*L), and 5 *µ*L of TaqMan® Universal PCR Master Mix (Applied Biosystems®, Foster City, CA, USA). Cycling parameters were 50°C for 2 minutes, 95°C for 10 minutes, followed by 45 cycles of 95°C for 15 seconds and 60°C for 1 minute. Quantification of bacterial DNA was done by comparison with a standard curve generated for each bacterial strain, diluted in a 10-fold series (*S. aureus*, *S. pneumoniae*, *H. influenzae*, *M. catarrhalis*, and *P. aeruginosa*), thus allowing for determination of bacterial loads.

### 2.3. Statistical Analysis

Comparisons between groups were made by Fisher's exact test, and agreement among the three specimens (AD, PT, and NPW) was assessed by kappa coefficients. In all analyses, *p* < 0.05 was considered statistically significant.

### 2.4. Ethics Statement

All legal guardians signed informed consent forms. The study was performed in accordance with the Declaration of Helsinki and approved by the Ethics Committee of the University Hospital, Medical School of Ribeirão Preto, file number 10466/2008.

## 3. Results

### 3.1. Demographic Data

A total of 51 children were enrolled: 37 in the tonsillectomy group and 14 in the cochlear implant control group. In both groups, children ages were 2 to 12 years, with mean ages of 6 years and 4.1 years, respectively, in the tonsillectomy and control groups.

### 3.2. Bacterial Detection by Real-Time PCR

The overall rates of bacterial detection in all sampling sites (PT, AD, and NPW) were similar between patients with chronic adenotonsillitis and controls (Table 2). *H. influenzae* and *S. pneumoniae* were the most frequently detected bacteria in both groups, with frequencies around 80%. *P. aeruginosa* and *M. catarrhalis* were also similarly detected in both groups, approximately 50% of the patients. The least frequent bacterium in all samples was *S. aureus*, detected in approximately 15% in both groups.

### 3.3. Comparison of Bacteria Detection Rates and Bacterial Loads between Different Sampling Sites

To assess the agreement in bacteria rates between the different sample sites, kappa test was used (Table 3).

In the control group, the correlation between AD and PT was good for *H*. *influenzae* (kappa: 0.6829), but only moderate for *M. catarrhalis* (kappa: 0.4324). Correlations between NPW and AD (kappa: 0.5714) or PT (kappa = 0.4286) were also moderate for *M. catarrhalis*. There was lack of agreement between different sampling sites with regard to all other bacteria in this group of patients.

In the group of patients with chronic adenotonsillitis, kappa analyses revealed no correlation between bacteria rates in PT and AD (Table 3). Comparison between NPWs and PT revealed only moderate correlation for *M. catarrhalis* (kappa: 0.6273) and *S. pneumoniae* (0.5325), but not for the other bacteria. Similarly, comparisons between NPWs and AD revealed moderate correlations for *S. aureus* (0.5277) and *S. pneumoniae* (0.4873).

To further probe into differences in the magnitudes of bacterial colonization of different sites, quantitative values of bacterial loads were compared among those sites for both groups of patients (Table 4). No significant differences in bacterial loads were detected, what may be partially due to restricted sizes of some subgroups.

### 3.4. Bacteria Detection and Intensity of Tonsillar Hypertrophy

**Figure 1:**
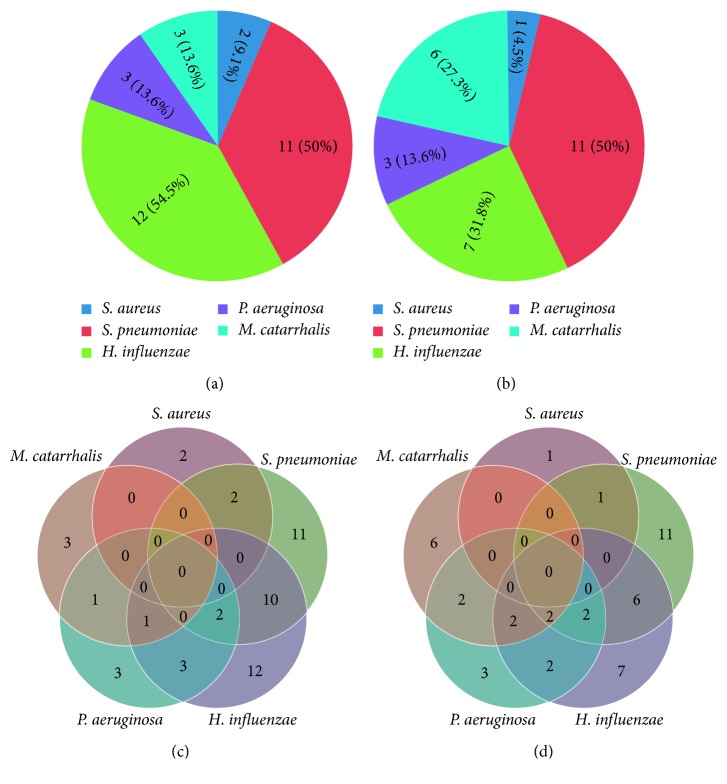
Bacterial detection in tonsils of patients with higher hypertrophy. The graph shows the frequency of bacterial detection in tonsils (a) and in adenoids (b). The graph shows the patients who presented bacterial codetection in the (c) tonsils and in adenoids (d).

Bacteria detection rates in different sampling sites were finally compared only among the 22 (59.4%) patients who had severe tonsillar hypertrophy (Brodsky index ≥3). *H. influenzae* was detected in palatine tonsils from 12 of those 22 patients (54.5%) and *S. pneumoniae* was detected in 11 (50%) patients (Figure 1(a)). The AD of highly hypertrophic patients were also frequently infected with *S. pneumoniae* and *H. influenza* (resp, 50% and 31.8%) (Figure 1(b)).

The frequencies of bacteria codetections were especially high in patients with more severe tonsillar hypertrophy (Figures 1(c) and 1(d)). More than one of the bacteria tested, and sometimes all four of them were codetected in 41% of tissue samples from PT and AD from patients with tonsillar hypertrophy (Figures 1(c) and 11(d)).

Kappa analyses were also performed considering only patients with severe tonsillar hypertrophy (Table 3). Remarkably, there was a good correlation (0.7179) between AD and NPW and moderate correlations between AD and PT (0.4086) and between PT and NPW (0.4211) with regard to detection of *M. catarrhalis*. With regard to *S. aureus*, *S. pneumoniae* and *H. influenzae*, the level of concordance between detection in severely hypertrophic palatine tonsils and in NPW was moderate.

## 4. Discussion

Chronic tonsillitis is characterized by chronic or recurrent infections of the tonsils and adenoids that can lead to tissue enlargement. Tonsillar hypertrophy is one of the most common diseases found in children and its etiology is still obscure. Its treatment is based on the administration of antibiotics and adeno/tonsillectomy, which is one of the most common surgical procedures performed worldwide. Many studies have reported that adeno/tonsillectomy leads to a reduction in the number of episodes of throat pain in children in the first year after surgery compared to nonsurgical treatment [1, 21–24].

Many studies have shown that the tonsils of patients with recurrent tonsillitis are colonized by a large number of bacteria, both pathogenic and nonpathogenic, showing that these agents possibly may play a role in the development of chronic tonsillar disease [1, 7, 21, 25]. In patients undergoing adenoidectomy, for instance, there is a decrease in the detection of pathogenic agents in the nasopharyngeal region [26].

There is evidence that large numbers of bacteria make up surface biofilms of the adenoids [27], supported by research 16s RNAs indicating that the tonsils are reservoirs of pathogens [28] hosting very diverse microbial communities, some of them potentially pathogenic. Confirming these data, Winther et al. observed a large number of bacteria located in adenoids, more specifically in the mucus that lines the surface of this tissue. In addition, bacterial biofilms were present in 8 of 9 adenoids and were more common in the caudal region of this tissue [27].

Pathogenic bacteria such as *Neisseria* sp., *Streptococcus* sp., *Haemophilus influenzae*, *Staphylococcus aureus*, *Actinomyces*, *Bacteroides*, *Prevotella, Porphyromonas*, *Peptostreptococcus*, *and Fusobacterium* sp. are often isolated from the nasopharynx, both from healthy and sick children [29]. Other studies, although the results were contradictory, some of the bacteria most commonly detected in the upper respiratory tract were also found in AD and PT of children with tonsillar hypertrophy, including *H. influenzae*, *M. catarrhalis*, and *S. pneumoniae* [11, 13, 15].

In the present study, detection and loading frequencies of genomic DNA were determined by real-time PCR for five of the most common bacteria of the nasopharyngeal microbiota, namely: *H. influenzae*, *M. catarrhalis*, *S. pneumoniae*, *P. aeruginosa*, and *S. aureus*. It is important to note that the tests were done on tissue fragments of both AD and PT, as well as on NPWs, which cannot distinguish the exact niche where these microbial genomic DNA were found. There is some evidence that points out that the microbiome of tonsils and adenoids may vary according to the microniche analyzed. Using *in situ* hybridization technique, for instance, Swidsinski et al. demonstrated in asymptomatic individuals that, depending on the type of bacteria, these microbes are mainly found either infiltrating the adenoid and tonsils, in the surface covered by a thick slime of inflammatory cells or within tissue macrophages [30].

Regardless of the state of tonsillar hypertrophy, the highest detection rates of *H. influenzae* and *S. pneumoniae* were found in approximately 80% of the patients, while *M. catarrhalis* and *P. aeruginosa* were found in almost 50% of the children, and *S. aureus* in only 15%. These results are in agreement with previously published data [31, 32], and corroborate with the findings that *H. influenzae*, *M. catarrhalis*, *S. pneumoniae*, and *P. aeruginosa* colonize the upper respiratory tract of a large proportion of children. In addition, the genome load for different bacteria varied significantly in the same tissue, suggesting that potentially pathogenic bacteria may vary widely in tonsillar biofilms.

The lack of uniform association of specific bacteria with tonsil hypertrophy is not surprising and suggests that the role of these bacteria in the development of tonsillar hypertrophy is far from clear and probably not due to a direct effect.

The sampling sites and methods used for bacterial detection are critical in this type of study. Pathogenic and anaerobic bacteria are easily detected in the nuclei of the tonsils, while commensal aerobic bacteria are found on the surface of these tissues [33, 34]. In addition, bacterial cultures detect only viable microorganisms, while molecular methods can detect scarce quantities of noncultured bacteria [35]. Also, molecular analyses based on sequencing and pirosequencing of 16S ribosomal RNA have been used with the objective to determine the microbiota localized on the surface and inside the adenoid tissue [36].

In the present study, different types of clinical samples were tested for five potentially pathogenic bacteria by a sensitive molecular assay, the real-time PCR assay. Nasopharyngeal lavages allowed the detection of planktonic bacteria, while fragments of tonsillar tissue provided direct association with sessile biofilms. No differences were observed in bacterial frequency or bacterial loads between control children and patients with adenotonsillar hypertrophy. We should emphasize that bacterial detection was widely different between adenoids and palatine tonsils in all patient groups. This suggests that there may be tissue-specific colonization by these bacteria in different tonsils, rather than a homogenous distribution of planktonic biofilm microorganisms in all tissues.

Several studies indicate that *H. influenzae* may be associated with tonsillar hypertrophy. For example, although Van Staaij and colleagues found similar rates of detection of *H. influenzae* in patients with and without chronic adenotonsillar disease, this bacterium was significantly more frequent in patients with more severe tonsillar hypertrophy. This result was in agreement with other published studies [37–39]. In addition, Brodsky et al. [32] found in *H. influenzae* a significant positive association between high bacterial load and tonsil weight.

In the present study, kappa analysis compared the detection of a bacterium at different sampling sites of different patient groups to assess whether extensive infection by certain bacteria could be associated with amygdala hypertrophy. In the control group, represented by the children as ATH group, despite the difference of slits in the middle ages, a good correlation was found between the detection of *H. influenzae* in the tonsils and in the adenoids, but the agreement was only moderate or poor for the detection of between other sampling sites and the other groups.

The rates of detection of *M. catarrhalis* were higher in patients with severe adenotonsillar hypertrophy, and there was good agreement between the detection of this bacterium in NPW, adenoids, and palatine tonsils of these patients. Perhaps *M. catarrhalis* infection may be relevant to the development of adenotonsillar hypertrophy or, alternatively, the involvement of *M. catarrhalis* in the normal nasopharynx microbiota is favored by tonsillar hypertrophy. In fact, the presence of MCR_1483, a protein generally expressed during inflammation of the respiratory tract, may facilitate colonization by *M. catarrhalis* [40].

The role of colonizing bacteria in the development of tonsillar hypertrophy is currently unknown. In our study, we evaluated a single facet of a complex interaction between the microbiota and the host. The influence of other factors, including the individual characteristics of each patient's immune system, coinfection, and other nonmicrobial agents, could eventually interfere with the results [41–43]. A particularly useful example of mutual cross protection in complex populations of bacteria was that *H. influenzae* could be protected from death mediated by complement by *M. catarrhalis* [41].

## 5. Conclusions

In summary, in the present study, a high proportion of children with chronic tonsillar inflammation and hypertrophy had detectable potentially pathogenic bacteria in tonsillar tissues, as well as in the control patients. Importantly, in patients with more severe tonsillar hypertrophy, there was good agreement between sites for detection of *M. catarrhalis*, suggesting that this bacterium may be associated with more severe tonsillar hypertrophy or, alternatively, the microenvironment of chronically inflamed hypertrophic tonsils can facilitate colonization by *M. catarrhalis.* The roles played by *M. catarrhalis* in the biofilm of severely hypertrophic tonsils may well be related to protection of certain other bacteria from innate immunity factors, in a way similar to what was documented for *H. influenza*e. More comprehensive prospection of tonsillar microbiomes are required in order to better assess the roles that bacteria may play in adenotonsillar hypertrophy.

## Figures and Tables

**Table 1:** Primers and probes used for bacterial detection.

Bacteria	Primers	Probes	References
*S. pneumoniae*	5′TGCAGAGCGTCCTTTGGTCTAT3′ (FORWARD)	FAM 5′TGGCGCCCATAAGCAACACTCGAA-Tamra 3′ TAMRA	Corless et al., [16]
	5′CTCTTACTCGTGGTTTCCAACTTGA3′ (REVERSE)		
*S. aureus*	5′GTTGCTTAGTGTTAACTTTAGTTGTA 3′ (FORWARD)	5′-VIC-AAGTCTAAGTAGCTCAGCAAATGCA-MGB-3′	Kilic et al., [17]
	5′AATGTCGCAGGTTCTTTATGTAATTT 3′ (REVERSE)		
*P. aeruginosa*	5′CGAGTACAACATGGCTCTGG 3′ (FORWARD)	5′-FAM-CCTGCAGCACCAGGTAGCGC-Tamra-3′	Feizabadi et al., [18]
	5′ACCGGACGCTCTTTACCATA 3′ (REVERSE)		
*H. influenzae*	5′CCAGCTGCTAAAGTATTAGTAGAAG 3′ (FORWARD)	5′-VIC-CAGATGCAGTTGAAGGTTATTTAG-MGB-3′	Abdeldaim et al., [19]
	5′TTCACCGTAAGATACTGTGCC 3′ (REVERSE)		
*M. catarrhalis*	5′GTCAAACAGCTGGAGGTATTGC 3′ (FORWARD)	5′-NED-ATCGCAATTGCAACTTT-MGB-3′	Heiniger et al., [20]
	5′GACATGATGCTCACCTGCTCTA 3′ (REVERSE)		

*S. pneumonia*: *Streptococcus pneumoniae*; *S. aureus*: *Staphylococcus aureus*; *P. aeruginosa*: *Pseudomonas aeruginosa*; *H. influenzae*: *Haemophilus influenzae*; *M. catarrhalis*: *Moraxella catarrhalis*.

**Table 2:** Detection rates of bacteria in tonsillar tissues and nasopharyngeal washes from patients with adenotonsillar hypertrophy and controls.

Bacteria	Palatine tonsils		Adenoids		NPW		Total	
	Controls *n* (%)	ATH *n* (%)	Controls *n* (%)	ATH *n* (%)	Controls *n* (%)	ATH *n* (%)	Controls *n* (%)	ATH *n* (%)
*S. aureus*	0 (0)	3 (8.1)	2 (14.2)	1 (2.7)	0 (0)	4 (10.8)	2 (14.2)	6 (16.2)
*S. pneumoniae*	4 (28.6)	19(51.3)	9 (64.2)	21(56.7)	11 (78.5)	26 (70.2)	12 (85.7)	29 (78.3)
*H. influenzae*	8 (57.1)	24(64.8)	9 (64.2)	16(43.2)	10 (71.4)	26(70.2)	12 (85.7)	30 (81)
*P. aeruginosa*	4 (28.6)	6 (16.2)	2 (14.2)	8 (21.6)	2 (14.2)	9 (24.3)	6 (42.8)	17 (45.9)
*M. catarrhalis*	3 (21.4)	6 (16.2)	4 (28.5)	15(40.5)	7 (50)	20 (54)	7 (50)	21 (56.7)

NPW: nasopharyngeal washes; ATH: adenotonsillar hypertrophy. *S. aureus*: *p* > 1.00; *S. pneumoniae*: *p* > 0.70; *H. influenzae*: *p* > 1.00; *P. aeruginosa*: *p* > 1.00; *M. catarrhalis*: *p* > 0.75. *S. pneumonia*: *Streptococcus pneumoniae*; *S. aureus*: *Staphylococcus aureus*; *H. influenzae*: *Haemophilus influenzae*; *P. aeruginosa*: *Pseudomonas aeruginosa*; *M. catarrhalis*: *Moraxella catarrhalis*.

**Table 3:** Analyses of agreement of bacteria detection rates among different specimens collected from patients with chronic adenotonsillitis, severe adenotonsillar hypertrophy, and controls.

Bacteria	Specimens	Patients	Kappa coefficient	95% CI
*S. aureus*	Adenoid × palatine tonsil	Control	0	(−1.04; 1.04)
		Chronic adenotonsillitis	−0.0435	(−0.1098; 0.0228)
		Severe adenotonsillar hypertrophy	−0.0769	(−0.1958; 0.0419)
	Palatine tonsil × nasopharyngeal wash	Control	0	(−1.04; 1.04)
		Chronic adenotonsillitis	−0.0465	(−0.1218; 0.0287)
		Severe adenotonsillar hypertrophy	−0.0769	(−0.1958; 0.0419)
	Adenoid × nasopharyngeal wash	Control	0	(−1.04; 1.04)
		Chronic adenotonsillitis	0.5277	(0.0599; 0.9954)
		Severe adenotonsillar hypertrophy	0.614	(0.1266; 1.0000)
	Adenoid × palatine tonsil	Control	−0.1455	(−0.5712; 0.2802)
		Chronic adenotonsillitis	0.132	(−0.1858; 0.4497)
		Severe adenotonsillar hypertrophy	0.0833	(−0.3336; 0.5003)
	Palatine tonsil × nasopharyngeal wash	Control	0.3171	(−0.1899; 0.8241)
*S. pneumoniae*		Chronic adenotonsillitis	0.5325	(0.2658; 0.7991)
		Severe adenotonsillar hypertrophy	0.5299	(0.1836; 0.8763)
	Adenoid × nasopharyngeal wash	Control	0.1967	(−0.0539; 0.4473)
		Chronic adenotonsilliti	0.4873	(0.2219; 0.7528)
		Severe adenotonsillar hypertrophy	0.5299	(0.1836; 0.8763)
	Adenoid × palatine tonsil	Control	0.6829	(0.2996; 1.0000)
		Chronic adenotonsillitis	0.2725	(−0.0046; 0.5495)
		Severe adenotonsillar hypertrophy	0.3937	(0.0567; 0.7307)
	Palatine tonsil × nasopharyngeal wash	Control	0.4179	(−0.1244; 0.9602)
*H. influenza*		Chronic adenotonsillitis	0.2825	(0.0243; 0.5408)
		Severe adenotonsillar hypertrophy	0.1538	(−0.1936; 0.5013)
	Adenoid × nasopharyngeal wash	Control	0.087	(−0.4156; 0.5895)
		Chronic adenotonsillitis	0.3854	(0.0724; 0.6984)
		Severe adenotonsillar hypertrophy	0.5217	(0.1575; 0.8859)
	Adenoid × palatine tonsil	Control	−0.2353	(−0.4760; 0.0054)
		Chronic adenotonsillitis	0.2986	(−0.0725; 0.6696)
		Severe adenotonsillar hypertrophy	0.2281	(−0.3034; 0.7646)
	Palatine tonsil × nasopharyngeal wash	Control	−0.1667	(−0.3277; −0.0056)
*P. aeruginosa*		Chronic adenotonsillitis	0.0082	(−0.3152; 0.3317)
		Severe adenotonsillar hypertrophy	0.0494	(−0.3519; 0.4507)
	Adenoid × nasopharyngeal wash	Control	0.1765	(−0.3573; 0.7103)
		Chronic adenotonsillitis	−0.0761	(−0.3555; 0.2034)
		Severe adenotonsillar hypertrophy	−0.2222	(−0.4076; −0.0369)
	Adenoid × palatine tonsil	Control	0.4324	(−0.1001; 0.9649)
		Chronic adenotonsillitis	0.3183	(0.0437; 0.5928)
		Severe adenotonsillar hypertrophy	0.4086	(−0.0008; 0.8180)
	Palatine tonsil × nasopharyngeal wash	Control	0.5714	(0.1830; 0.9598)
*M. catarrhalis*		Chronic adenotonsillitis	0.6273	(0.3876; 0.8671)
		Severe adenotonsillar hypertrophy	0.7179	(0.4334; 1.0000)
	Adenoid × nasopharyngeal wash	Control	0.4286	(0.0402; 0.8170)
		Chronic adenotonsillitis	0.2825	(0.0761; 0.4890)
		Severe adenotonsillar hypertrophy	0.4211	(0.0989; 0.7432)

*S. pneumonia*: *Streptococcus pneumoniae*; *S. aureus*: *Staphylococcus aureus*; *H. influenzae*: *Haemophilus influenzae*; *P. aeruginosa*: *Pseudomonas aeruginosa*; *M. catarrhalis*: *Moraxella catarrhalis.*

**Table 4:** Descriptive statistics for bacterial loads by group and tissue.

Tissue	Bacteria	Mean	Standard deviation	Minimum	1° Quartile	Median	3° Quartile	Maximum
*Adenotonsillitis*								
Adenoid	*S. aureus*	16.64	0.56	15.89	16.28	16.7	16.99	17.25
	*S. pneumoniae*	17.82	1.9	13.31	16.87	17.84	19.15	21.28
	*H. influenza*	10.65	2.31	5.95	9.34	10.86	12.44	14.33
	*P. aeruginosa*	8.95	1.12	7.01	8.54	9.05	9.99	10.05
	*M. catarrhalis*	11.59	0.95	10.88	11.02	11.16	11.95	13.36
	*S. aureus*	17.58		17.58	17.58	17.58	17.58	17.58
	*S. pneumoniae*	18.13	1.95	12.61	16.95	18.47	19.11	21.31
	*H. influenza*	10.41	1.59	7.29	9.04	10.47	11.53	13.36
	*P. aeruginosa*	7.7	0.6	6.66	7.2	7.9	8.23	8.24
Palatine tonsil	*M. catarrhalis*	13.05	1.68	10.67	11.26	13.38	14.05	16.6
	*S. aureus*	14.87	0.51	14.11	14.57	15.09	15.17	15.18
	*S. pneumoniae*	17.62	6	6.49	14.14	17.44	19.59	39.47
	*H. influenza*	10.31	2.37	3.91	8.76	10.47	11.83	14.21
	*P. aeruginosa*	4.77	1.37	1.24	4.92	5	5.19	5.85
Nasopharyngeal washes	*M. catarrhalis*	12.97	3.36	7.38	9.77	13.53	15.36	18.85
	*S. aureus*	^*∗*^	^*∗*^	^*∗*^	^*∗*^	^*∗*^	^*∗*^	^*∗*^
	*S. pneumoniae*	21.99	4.57	19.48	19.55	19.82	24.43	28.84
	*H. influenza*	11.33	2.22	8.06	9.76	11.31	12.67	15.07
	*P. aeruginosa*	8.62	1.33	6.82	7.72	8.81	9.51	10.02
Adenoid	*M. catarrhalis*	14.91	1.07	13.74	13.74	15.18	15.82	15.82
	*S. aureus*	17.51	0.52	17.14	17.14	17.51	17.87	17.87
	*S. pneumoniae*	20.83	5.22	15.07	16.78	20.86	22.36	31.61
	*H. influenza*	11.3	2.43	9.01	9.71	10.63	12.15	16.8
	*P. aeruginosa*	7.05	3.09	4.86	4.86	7.05	9.23	9.23
Palatine tonsil	*M. catarrhalis*	13.88	1.88	12.43	12.62	13.24	15.13	16.59
	*S. aureus*	^*∗*^	^*∗*^	^*∗*^	^*∗*^	^*∗*^	^*∗*^	^*∗*^
	*S. pneumoniae*	18.04	3.74	12.81	14.71	18.1	20.54	24.63
	*H. influenza*	9.8	2.82	4.02	9.01	9.82	11.22	13.9
	*P. aeruginosa*	4.89	0.3	4.67	4.67	4.89	5.1	5.1
*Controls*								
Nasopharyngeal washes	*M. catarrhalis*	14.28	5.21	8.09	10.36	11.51	19.38	21.31

^*∗*^The bacteria were not detected in the specific sample. *S. pneumonia*: *Streptococcus pneumoniae*; *S. aureus: Staphylococcus aureus*; *H. influenzae*: *Haemophilus influenzae*; *P. aeruginosa*: *Pseudomonas aeruginosa*; *M. catarrhalis*: *Moraxella catarrhalis*.

## Data Availability

The data used to support the findings of this study are included within the article.
